# Multidimensional Associations Between Psychiatric Symptoms, Sleep Disturbance, Biological Variables, and Fibromyalgia Severity: A Classification and Regression Tree and Path Analysis Study

**DOI:** 10.3390/jcm15166143

**Published:** 2026-08-07

**Authors:** Selçuk Akan, Mustafa Uğurlu, Bahadır Ertürk

**Affiliations:** 1 Department of Internal Medicine, Physical Medicine and Rehabilitation Hospital, Ankara Bilkent City Hospital, Ankara 06800, Türkiye; 2 Department of Psychiatry, Ankara Bilkent City Hospital, Yıldırım Beyazıt University, Ankara 06800, Türkiye; dr_ugurlu@yahoo.com; 3 Department of Family Medicine, Kaman District Health Directorate, Kırşehir 40300, Türkiye; bahadirerturk@gmail.com

**Keywords:** fibromyalgia, depression, anxiety, sleep disturbance, biopsychosocial model, thyroid function, vitamin D

## Abstract

**Background**: Fibromyalgia (FM) is a complex chronic pain disorder characterized by interactions among psychological, biological, and clinical factors. Although psychiatric symptoms and sleep disturbance are recognized contributors to disease burden, their multidimensional relationships with selected biological variables and fibromyalgia severity remain incompletely understood. **Objective**: This study aimed to investigate multidimensional associations among psychiatric symptoms, sleep disturbance, selected biological variables, and fibromyalgia severity using complementary analytical approaches, including multiple linear regression, Classification and Regression Tree (CART) analysis, and path analysis. **Methods**: In this cross-sectional study, 102 women with FM and 53 healthy female controls underwent standardized clinical and laboratory assessment. Fibromyalgia severity was assessed using the Fibromyalgia Impact Questionnaire (FIQ), depressive symptoms with the Beck Depression Inventory (BDI), anxiety symptoms with the Beck Anxiety Inventory (BAI), and sleep disturbance with the Jenkins Sleep Scale (JSS). Serum thyroid-stimulating hormone (TSH) and 25-hydroxyvitamin D concentrations were measured as selected biological variables. Multiple linear regression identified independent associations with disease severity, CART analysis evaluated hierarchical classification patterns, and path analysis examined direct and indirect associations within a predefined conceptual framework. **Results**: Depressive symptoms, sleep disturbance, and anxiety symptoms were independently associated with greater fibromyalgia severity, whereas TSH and vitamin D showed no consistent independent associations. CART identified sleep disturbance as the primary hierarchical classifier, followed by depressive and anxiety symptoms, achieving an overall classification accuracy of 93.5% (AUC = 0.951). Within the proposed conceptual framework, path analysis demonstrated direct associations between psychological symptoms and fibromyalgia severity, whereas TSH and vitamin D exhibited distinct indirect relationships through depressive and anxiety symptoms, respectively. **Conclusions**: Psychological symptoms were more consistently associated with fibromyalgia severity than the selected biological variables across complementary analytical approaches. The integrated use of multiple linear regression, CART analysis, and path analysis provided a multidimensional perspective on fibromyalgia and highlighted the importance of comprehensive biopsychosocial assessment while emphasizing that the observed findings represent statistical associations rather than causal relationships.

## 1. Introduction

Fibromyalgia (FM) is a chronic pain syndrome characterized by widespread musculoskeletal pain accompanied by a broad spectrum of symptoms, including fatigue, cognitive complaints, sleep-related disturbances, and emotional distress. Rather than representing a disorder driven by a single pathological mechanism, FM is currently regarded as a heterogeneous condition arising from complex interactions among neurobiological, psychological, and social processes [1,2]. This multifaceted nature contributes to substantial interindividual variability in symptom severity, functional impairment, and treatment response, making comprehensive clinical assessment challenging. Accordingly, understanding variations in disease severity requires an integrated perspective that considers the interplay of biological, psychological, and behavioral dimensions rather than focusing on isolated mechanisms.

Among the factors contributing to the clinical heterogeneity of FM, psychological symptoms and sleep disturbance have consistently emerged as key correlates of symptom burden and functional impairment. Depression and anxiety are highly prevalent in individuals with FM and are associated with greater pain intensity, poorer physical functioning, and reduced health-related quality of life [2,3]. Increasing evidence also indicates that frequent sleep-related complaints are closely intertwined with pain perception, emotional regulation, and cognitive functioning [4]. Rather than representing isolated manifestations, these domains appear to interact through reciprocal mechanisms involving central sensitization, dysregulation of pain modulation, and altered stress responses. Recent studies further suggest that psychological variables should be considered as interconnected components of a multidimensional symptom network rather than as independent contributors, reinforcing the need for integrated analytical approaches when investigating disease severity in FM [5,6,7].

Although psychological factors constitute a major component of FM symptomatology, growing evidence suggests that biological processes may also contribute to individual differences in clinical presentation. Among the numerous biological factors investigated, thyroid function and vitamin D status have attracted particular interest because of their reported associations with fatigue, mood disturbances, cognitive symptoms, and chronic pain [8,9,10,11]. However, the available evidence remains inconclusive, with studies reporting inconsistent findings regarding their relationship with FM severity. Rather than serving as disease-specific biomarkers, these variables may influence symptom expression indirectly through their interactions with psychological and neurophysiological processes. Based on this rationale, thyroid-stimulating hormone (TSH) and vitamin D were selected as representative biological variables to explore their potential contributions within an integrated biopsychosocial model.

Despite the growing body of literature on psychological and biological correlates of FM, the complex interplay among these domains remains insufficiently characterized. Most previous investigations have focused on isolated associations or conventional regression-based analyses, which may not fully capture the multidimensional relationships underlying disease heterogeneity. Consequently, distinguishing clinically relevant classification patterns from the broader network of symptom interactions remains challenging when these analytical approaches are applied in isolation. Considering that FM symptoms emerge through the interaction of multiple interconnected processes rather than independent pathways, analytical strategies capable of identifying clinically meaningful patterns and structural relationships may provide additional insights beyond traditional statistical approaches. In this context, Classification and Regression Tree (CART) analysis offers an intuitive framework for identifying clinically relevant classification patterns, whereas path analysis enables the evaluation of hypothesized relationships among biological, psychological, and clinical variables within a unified conceptual model [12]. Accordingly, integrating these complementary analytical approaches may facilitate a more comprehensive understanding of the multidimensional factors associated with fibromyalgia severity.

Against this background, the present study sought to examine the relationships among psychiatric symptoms, sleep disturbance, selected biological variables, and fibromyalgia severity within a biopsychosocial framework. Specifically, we aimed to (i) explore clinically relevant classification patterns that distinguish women with FM from healthy controls using Classification and Regression Tree (CART) analysis and (ii) investigate the direct and indirect relationships among psychological, biological, and clinical variables through path analysis. By integrating these complementary analytical approaches, we sought to provide a multidimensional perspective on factors associated with disease severity rather than to infer causal mechanisms or establish diagnostic models.

## 2. Materials and Methods

### 2.1. Study Design and Participant Selection

This cross-sectional observational case–control study was conducted to investigate the multidimensional associations among psychiatric symptoms, sleep disturbance, selected biological variables, and fibromyalgia severity in women with fibromyalgia. A total of 102 women diagnosed with fibromyalgia (FM) and 53 healthy female controls aged 18–65 years were consecutively recruited from the outpatient clinic of the Department of Physical Medicine and Rehabilitation at Ankara Bilkent City Hospital between May 2022 and August 2023. The diagnosis of FM was established according to the 2016 revised American College of Rheumatology (ACR) classification criteria [1].

Only women were included because fibromyalgia predominantly affects females, and restricting the study population to one sex was intended to reduce biological heterogeneity and minimize potential confounding related to sex-specific hormonal influences, pain perception, and psychological symptom expression. Although this approach enhanced cohort homogeneity and internal validity, it may limit the generalizability of the findings to male patients with FM.

Participants were excluded if they had severe psychiatric disorders requiring hospitalization, active neurological disease, endocrine disorders known to affect thyroid function, malignancy, pregnancy or lactation, or were receiving vitamin D or thyroid hormone supplementation.

The study was approved by the Local Ethics Committee of Ankara Bilkent City Hospital (Approval No. E2-22-1282; 19 January 2022) and was conducted in accordance with the Declaration of Helsinki. Written informed consent was obtained from all participants before enrollment.

### 2.2. Clinical and Laboratory Assessment

All participants underwent a standardized clinical evaluation that included assessment of fibromyalgia severity, depressive symptoms, anxiety symptoms, and sleep disturbance.

Fibromyalgia severity was assessed using the Fibromyalgia Impact Questionnaire (FIQ), a validated instrument that evaluates the overall impact of FM on physical functioning, symptoms, and health-related quality of life. Higher FIQ scores indicate greater disease impact [13].

Depressive symptoms were assessed using the Beck Depression Inventory (BDI), which has demonstrated satisfactory validity and reliability in Turkish populations [14,15]. Anxiety symptoms were evaluated using the Beck Anxiety Inventory (BAI), which has established psychometric validity in the Turkish population [16].

Sleep disturbance was evaluated using the Jenkins Sleep Scale (JSS), a brief self-report instrument designed to assess the frequency of common sleep-related complaints experienced during the preceding month [17]. Consistent with the construct measured by the JSS, the term sleep disturbance is used throughout this manuscript rather than sleep quality. Higher scores indicate more frequent sleep-related disturbances.

Serum thyroid-stimulating hormone (TSH) and 25-hydroxyvitamin D [25(OH)D] concentrations were measured as the selected biological variables of interest. These variables were chosen because of their reported associations with chronic pain, fatigue, mood disturbances, and fibromyalgia symptom expression and were evaluated as potential biological correlates within the biopsychosocial framework of the study rather than as disease-specific biomarkers [8,9,10,11].

### 2.3. Analytical Framework

To address the multidimensional nature of fibromyalgia (FM), we designed an analytical strategy that combined two complementary statistical approaches, each serving a distinct methodological objective. Given the complex interplay among psychological symptoms, sleep disturbance, selected biological variables, and disease severity, a single analytical method was considered insufficient to fully characterize these relationships.

Classification and Regression Tree (CART) analysis, based on recursive partitioning, was employed as an exploratory classification method to identify hierarchical patterns that distinguished women with FM from healthy controls. In contrast, path analysis was used to evaluate hypothesized direct and indirect associations among psychological symptoms, selected biological variables, and fibromyalgia severity within a predefined conceptual framework.

These analytical approaches were considered complementary because CART is well suited to identifying clinically relevant classification patterns and complex variable interactions, whereas path analysis enables simultaneous evaluation of multiple direct and indirect associations among observed variables. By integrating these methods, we aimed to obtain a more comprehensive understanding of multidimensional associations related to fibromyalgia severity rather than to establish causal or temporal relationships.

### 2.4. CART Model Development and Performance Evaluation

Classification and Regression Tree (CART) analysis was performed to identify hierarchical classification patterns that distinguished women with fibromyalgia (FM) from healthy controls. CART was selected because it can model complex, non-linear interactions among predictor variables while generating clinically interpretable decision rules through recursive partitioning.

The final CART model was developed using the Gini impurity criterion with balanced class weights to approximate equal prior probabilities. Tree complexity was constrained by a maximum tree depth of three levels and a minimum terminal node size of five observations. Candidate predictor variables included depressive symptoms (BDI), anxiety symptoms (BAI), and sleep disturbance (JSS). Recursive partitioning automatically selected the variables and decision thresholds that maximized classification performance, resulting in a hierarchical decision tree.

Model performance was evaluated on the complete study dataset using the confusion matrix together with sensitivity, specificity, positive predictive value (PPV), negative predictive value (NPV), overall accuracy, and the area under the receiver operating characteristic curve (AUC), with 95% confidence intervals reported where applicable. Because the CART model was applied as an exploratory classification method and no external validation in an independent cohort was performed, the identified decision thresholds should be interpreted as sample-specific findings requiring independent external validation before clinical application.

### 2.5. Path Model Specification and Estimation

Path analysis was performed to examine the direct and indirect associations among psychological symptoms, selected biological variables, and fibromyalgia severity within a predefined biopsychosocial framework. The model was specified a priori based on theoretical considerations and previous evidence regarding the relationships among thyroid function, vitamin D status, psychological symptoms, sleep disturbance, and FM severity. Because of the modest sample size, a parsimonious model was intentionally constructed to evaluate the principal hypothesized pathways rather than to represent the full complexity of FM pathophysiology.

The model was estimated using maximum likelihood estimation in IBM SPSS AMOS (Version 26.0). Model fit was evaluated using multiple complementary goodness-of-fit indices, including the chi-square statistic (χ^2^), Comparative Fit Index (CFI), Goodness-of-Fit Index (GFI), Standardized Root Mean Square Residual (SRMR), and Root Mean Square Error of Approximation (RMSEA). Standardized path coefficients (β), together with their standard errors, 95% confidence intervals, and corresponding *p* values, were used to quantify the strength of the hypothesized associations. Indirect associations were evaluated using bootstrap estimation with bias-corrected 95% confidence intervals. Because the study employed a cross-sectional design, all pathways were interpreted as statistical associations within the proposed conceptual framework rather than as evidence of causal or temporal relationships.

### 2.6. Statistical Analysis

All statistical analyses were performed using IBM SPSS Statistics version 26.0 (IBM Corp., Armonk, NY, USA). Path analysis was conducted using IBM SPSS AMOS version 26.0. Continuous variables were examined for normality using the Shapiro–Wilk test together with visual inspection of histograms and Q–Q plots. Normally distributed variables are presented as mean ± standard deviation (SD), whereas non-normally distributed variables are summarized as median (interquartile range [IQR]). Categorical variables are presented as frequencies and percentages.

Between-group comparisons were performed using the independent-sample *t*-test or the Mann–Whitney U test, as appropriate. Categorical variables were compared using the chi-square test or Fisher’s exact test. Multiple linear regression analyses were performed to evaluate independent associations between clinical and biological variables and fibromyalgia severity. Additional regression models adjusted for age and body mass index (BMI) were constructed to assess the robustness of the findings.

For the CART analysis, model performance was evaluated using sensitivity, specificity, positive predictive value (PPV), negative predictive value (NPV), overall accuracy, and the area under the receiver operating characteristic curve (AUC), together with corresponding 95% confidence intervals. All statistical tests were two-sided, and a *p* value < 0.05 was considered statistically significant.

## 3. Results

### 3.1. Participant Characteristics

A total of 155 women were included in the study, comprising 102 patients with fibromyalgia (FM) and 53 healthy controls. Psychiatric symptom and sleep disturbance scores are presented in Table 1, and hematologic and biochemical findings are summarized in Table 2. Compared with healthy controls, women with FM had significantly higher depressive symptoms, anxiety symptoms, and sleep disturbance scores (Table 1). Among the laboratory variables evaluated, RBC and free thyroxine (fT4) levels were significantly lower in the FM group, whereas no significant between-group differences were observed for TSH, 25-hydroxyvitamin D, or the remaining hematologic and biochemical variables (Table 2).

### 3.2. Multiple Linear Regression Analysis

Multiple linear regression analysis was performed to identify independent associations between clinical and biological variables and fibromyalgia severity among women with FM. The overall regression model was statistically significant (R^2^ = 0.533, adjusted R^2^ = 0.509, F = 21.921, *p* < 0.001). Depressive symptoms (standardized β = 0.374, *p* < 0.001), sleep disturbance (standardized β = 0.338, *p* < 0.001), and anxiety symptoms (standardized β = 0.246, *p* = 0.003) were independently associated with higher FIQ scores. In contrast, serum TSH (standardized β = 0.083, *p* = 0.255) and 25-hydroxyvitamin D concentrations (standardized β = 0.119, *p* = 0.109) were not independently associated with fibromyalgia severity.

To evaluate the robustness of these findings, an additional regression model adjusted for age and body mass index (BMI) was constructed. The adjusted model also remained statistically significant (R^2^ = 0.541, adjusted R^2^ = 0.507, F = 15.839, *p* < 0.001). The associations of depressive symptoms, anxiety symptoms, and sleep disturbance with fibromyalgia severity remained statistically significant after adjustment, whereas TSH, vitamin D, age, and BMI were not independently associated with FIQ scores.

### 3.3. Classification and Regression Tree Analysis

Classification and Regression Tree (CART) analysis identified sleep disturbance, depressive symptoms, and anxiety symptoms as the principal variables contributing to the hierarchical classification of women with fibromyalgia (FM) and healthy controls. Sleep disturbance, assessed using the Jenkins Sleep Scale (JSS), constituted the root node of the decision tree with an optimal threshold of 10.5 points. Among participants with JSS scores ≤ 10.5, depressive symptoms (BDI) and subsequently anxiety symptoms (BAI) provided additional hierarchical discrimination between the two groups.

The CART model identified sleep disturbance, assessed using the Jenkins Sleep Scale (JSS), as the primary splitting variable, followed by depressive symptoms (BDI) and anxiety symptoms (BAI). The tree illustrates the hierarchical classification structure generated by recursive partitioning. Model performance is summarized in Table 3, and the corresponding confusion matrix is presented in Table 4. The identified decision thresholds should be considered exploratory, as they are sample-specific and require external validation in independent cohorts before clinical application.

**Figure 1:**
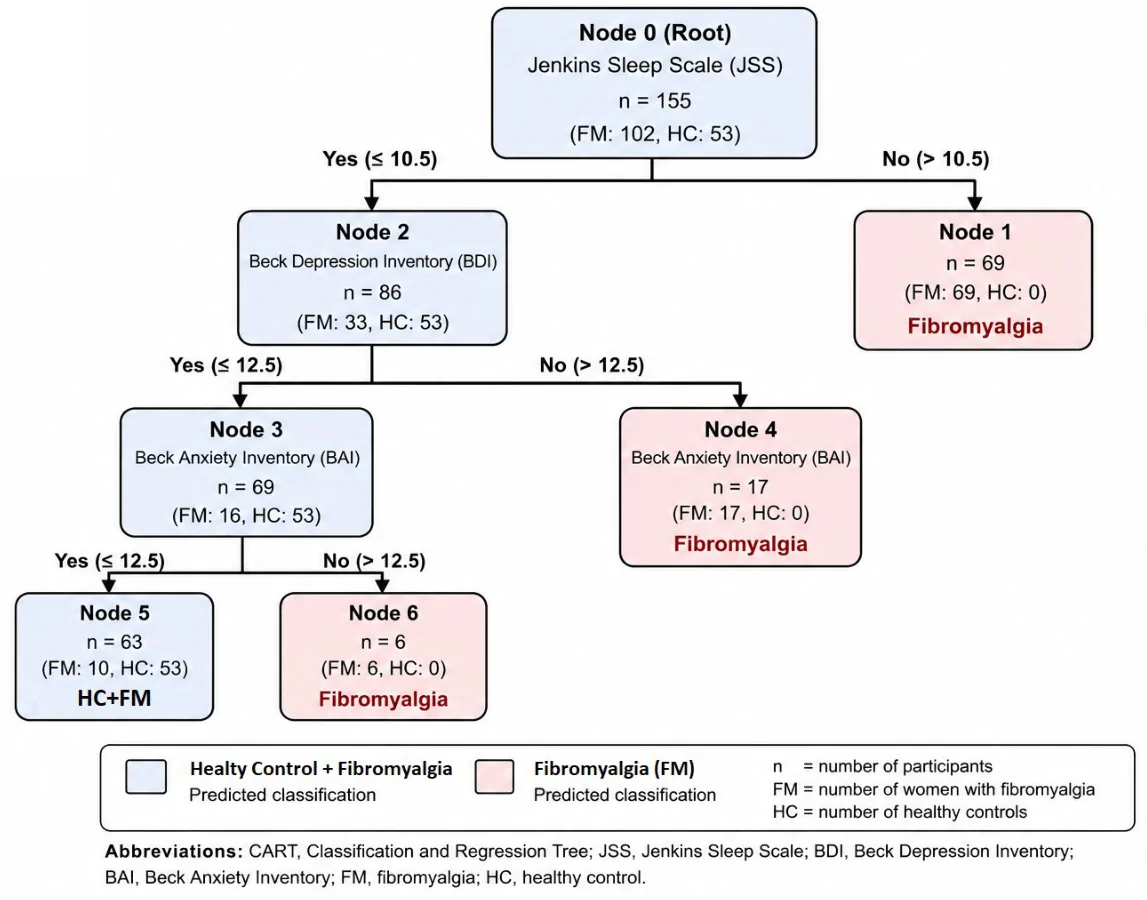
Classification and Regression Tree (CART) model distinguishing women with fibromyalgia from healthy controls.

The final CART model correctly classified 145 of 155 participants, corresponding to an overall accuracy of 93.5%. Sensitivity was 90.2% (95% CI, 82.9–94.6%), specificity was 100% (95% CI, 93.2–100%), positive predictive value (PPV) was 100%, negative predictive value (NPV) was 84.1%, and the area under the receiver operating characteristic curve (AUC) was 0.951. The resulting decision tree is presented in Figure 1. The corresponding classification performance metrics are summarized in Table 3, and the confusion matrix is presented in Table 4, demonstrating that 92 of 102 patients with FM and all 53 healthy controls were correctly classified.

Performance metrics were calculated using the final CART model. Exact binomial 95% confidence intervals are presented where applicable.

### 3.4. Path Analysis

The predefined path model demonstrated an acceptable overall fit to the data according to multiple goodness-of-fit indices (Table 5).

**Figure 2:**
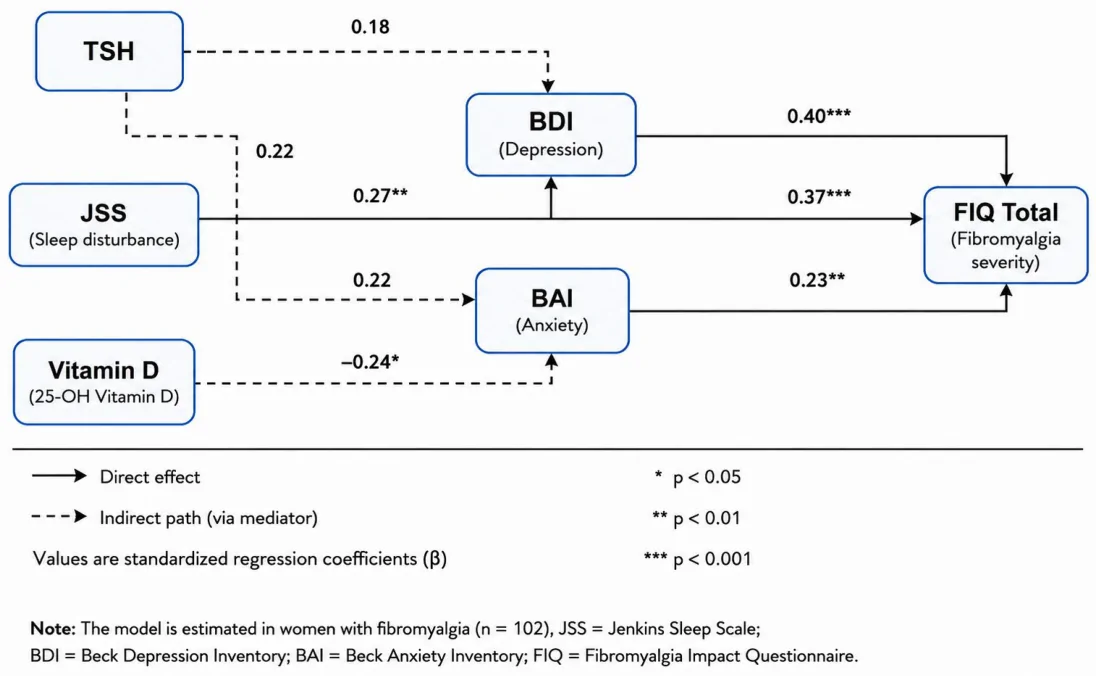
Path model illustrating the associations among thyroid function, vitamin D, sleep disturbance, psychiatric symptoms, and fibromyalgia impact in women with fibromyalgia. Abbreviations: BAI, Beck Anxiety Inventory; BDI, Beck Depression Inventory; FIQ, Fibromyalgia Impact Questionnaire; JSS, Jenkins Sleep Scale; TSH, thyroid-stimulating hormone.

The resulting path model is illustrated in Figure 2, and the corresponding standardized path coefficients are presented in Table 6.

Standardized path model showing the associations between thyroid-stimulating hormone (TSH), serum vitamin D, sleep disturbance (Jenkins Sleep Scale, JSS), depressive symptoms (Beck Depression Inventory, BDI), anxiety symptoms (Beck Anxiety Inventory, BAI), and fibromyalgia impact (Fibromyalgia Impact Questionnaire, FIQ Total) in women with fibromyalgia (*n* = 102). Solid arrows indicate direct associations with the outcome, whereas dashed arrows represent hypothesized intermediary pathways. Values on the arrows are standardized regression coefficients (β). The TSH–BDI pathway was of borderline statistical significance (β = 0.18, *p* = 0.065) and is therefore shown with a dashed arrow. The model is intended to illustrate associations within a cross-sectional framework and should not be interpreted as implying causality.

Among the direct associations with fibromyalgia severity, depressive symptoms had the largest standardized path coefficient (β = 0.399, *p* < 0.001), followed by sleep disturbance (β = 0.362, *p* < 0.001) and anxiety symptoms (β = 0.224, *p* = 0.005). Serum TSH showed a positive but non-significant association with depressive symptoms (β = 0.177, *p* = 0.065), whereas vitamin D was inversely associated with anxiety symptoms (β = −0.246, *p* = 0.013).

Bootstrap analysis supported a significant indirect association between sleep disturbance and fibromyalgia severity through depressive symptoms, as well as between vitamin D and fibromyalgia severity through anxiety symptoms. In contrast, the indirect association between TSH and fibromyalgia severity through depressive symptoms did not reach statistical significance because the bootstrap confidence interval included zero. The bootstrap estimates of the indirect effects are summarized in Table 7.

Statistical significance was determined according to the bias-corrected bootstrap 95% confidence interval. Indirect effects were considered statistically significant when the confidence interval did not include zero.

## 4. Discussion

### 4.1. Principal Findings

This study investigated multidimensional associations among psychiatric symptoms, sleep disturbance, selected biological variables, and fibromyalgia severity using complementary analytical approaches. Three principal findings emerged. First, depressive symptoms, sleep disturbance, and anxiety symptoms were consistently associated with greater fibromyalgia severity across multiple analytical methods, including multiple linear regression and path analysis. Second, serum TSH and 25-hydroxyvitamin D were not independently associated with fibromyalgia severity, although both showed indirect associations with psychological variables. Third, CART analysis identified sleep disturbance as the primary hierarchical classifier, followed by depressive and anxiety symptoms, highlighting the prominent contribution of psychological variables to the hierarchical classification of women with FM and healthy controls.

### 4.2. Interpretation of Psychological Findings

The most consistent finding of the present study was the close association of psychological variables with fibromyalgia severity across complementary analytical approaches. Depressive symptoms, sleep disturbance, and anxiety symptoms remained independently associated with FIQ scores in the multiple regression analyses, occupied central positions within the path model, and collectively formed the hierarchical classification structure identified by CART. The consistency of these findings across different statistical methods strengthens the interpretation that psychological symptom burden is closely associated with clinical disease severity in women with FM, although the cross-sectional design precludes conclusions regarding causality [2,3,4,5,6,7].

Among the psychological variables examined, sleep disturbance occupied a particularly prominent position by constituting the root node of the CART model. This finding suggests that sleep disturbance may represent the earliest hierarchical classifier within the present CART model, before additional psychological variables contribute to classification. However, this pattern should be interpreted as a data-driven classification result rather than evidence that sleep disturbance represents the primary pathogenic mechanism underlying FM. Bootstrap analysis further demonstrated a significant indirect association between sleep disturbance and fibromyalgia severity through depressive symptoms, reinforcing the central role of sleep disturbance within the multidimensional framework identified in this study. These observations are consistent with recent systematic reviews showing that sleep disturbance and depressive symptoms are closely interconnected and jointly contribute to symptom burden and functional impairment in FM [4,6,7].

Depressive symptoms demonstrated the strongest standardized association with fibromyalgia severity in both the regression and path analyses, followed by sleep disturbance and anxiety symptoms. This finding is consistent with previous studies reporting strong relationships between depressive symptom burden and functional impairment in FM [2,3]. Similar multidimensional relationships among pain, depressive symptoms, sleep disturbance, and functional impairment have also been demonstrated using structural equation modeling and network analysis [12]. Nevertheless, these psychological domains are likely to influence one another bidirectionally. Accordingly, the present findings should be interpreted as reflecting multidimensional clinical associations rather than unidirectional causal pathways.

Anxiety symptoms also remained independently associated with fibromyalgia severity, although their standardized association was smaller than that observed for depressive symptoms and sleep disturbance. This pattern suggests that anxiety represents an important, albeit comparatively less influential, component of the multidimensional psychological profile of FM. Furthermore, recent evidence indicates that psychological symptom profiles may also be influenced by underlying biological and genetic factors, underscoring the complexity of the biopsychosocial interactions underlying FM [18]. Future longitudinal studies are needed to clarify the temporal relationships among anxiety, depressive symptoms, sleep disturbance, and disease severity.

### 4.3. Interpretation of Biological Findings

Unlike the psychological variables, the selected biological variables were not independently associated with fibromyalgia severity after adjustment for the other variables included in the regression models. Neither serum TSH nor 25-hydroxyvitamin D concentrations remained significant independent correlates of FIQ scores, indicating that their direct contribution to disease severity was limited in the present study population. These findings are broadly consistent with previous studies evaluating thyroid function and vitamin D status in FM, which have reported heterogeneous and often inconsistent associations [8,9,10,11].

Although serum TSH showed a borderline positive association with depressive symptoms, bootstrap analysis did not support a statistically significant indirect association with fibromyalgia severity because the bias-corrected 95% confidence interval included zero. Accordingly, this finding should be interpreted cautiously as an exploratory observation rather than as evidence of a clinically meaningful mediation pathway.

Similarly, serum 25-hydroxyvitamin D was not independently associated with fibromyalgia severity in the regression analyses but demonstrated an inverse association with anxiety symptoms in the path model. Bootstrap analysis further supported a significant indirect association between vitamin D and fibromyalgia severity through anxiety symptoms, suggesting that any relationship between vitamin D and disease severity may be mediated, at least in part, through psychological pathways. However, given the cross-sectional design, this finding should be interpreted as exploratory and requires confirmation in prospective studies.

The observed reductions in RBC count and free thyroxine (fT4) levels should also be interpreted as exploratory findings because these variables were not incorporated into the structural model. Their potential clinical significance remains uncertain and requires confirmation in larger prospective and mechanistic studies.

Overall, the biological variables examined in this study demonstrated less consistent independent associations with fibromyalgia severity than the psychological variables. Rather than acting as direct correlates of disease severity, they may represent components of a broader biopsychosocial network in which biological and psychological processes interact.

### 4.4. Contribution of the Analytical Framework

An important strength of the present study was the integration of complementary statistical approaches to examine fibromyalgia from different analytical perspectives. Rather than relying on a single analytical technique, we combined multiple linear regression, CART analysis, and path analysis to address complementary research questions. This strategy enabled evaluation of independent associations, hierarchical classification patterns, and direct and indirect relationships within the same study population.

The findings were largely consistent across analytical approaches. Multiple linear regression identified depressive symptoms, sleep disturbance, and anxiety symptoms as independent correlates of fibromyalgia severity, CART analysis identified sleep disturbance as the primary hierarchical classifier distinguishing women with FM from healthy controls, and path analysis illustrated the interrelationships among psychological and selected biological variables within the proposed conceptual framework. Similar multidimensional relationships have recently been explored using structural equation modeling and network analysis, supporting the value of complementary analytical approaches for understanding the complexity of FM [12].

Nevertheless, these analytical approaches provide complementary perspectives on the same clinical phenomenon rather than independent confirmation of causal mechanisms. Because all analyses were based on cross-sectional data from a single cohort, the observed relationships should be interpreted as complementary statistical evidence of multidimensional clinical associations rather than as evidence of temporal or causal pathways.

### 4.5. Clinical Implications

The present findings have several potential clinical implications. The consistent associations observed between depressive symptoms, sleep disturbance, anxiety symptoms, and fibromyalgia severity emphasize the importance of comprehensive assessment in women with FM. Rather than focusing exclusively on pain intensity, routine clinical evaluation may benefit from the simultaneous assessment of psychological symptoms and sleep disturbance, as these domains appear to be closely related to overall disease burden [2,4,6,7].

The integrated analytical approach adopted in this study also illustrates how complementary statistical methods can provide additional clinical insights beyond conventional regression analyses alone. Although the decision thresholds identified by CART should not be interpreted as clinically validated diagnostic cut-off values, they may inform future research aimed at developing externally validated risk-stratification models.

### 4.6. Strengths and Limitations

This study has several strengths. To our knowledge, it is among the few studies to evaluate psychiatric symptoms, sleep disturbance, selected biological variables, and fibromyalgia severity within a single analytical framework. The integration of multiple linear regression, CART analysis, and path analysis enabled complementary evaluation of independent associations, hierarchical classification patterns, and direct and indirect relationships within the same cohort. Furthermore, all participants were assessed using standardized and validated clinical instruments, and the consistency of findings across different analytical approaches strengthens the robustness of the overall results.

Several limitations should also be acknowledged. First, the cross-sectional design precludes conclusions regarding causality or temporal relationships. Second, only women were included, which reduced biological heterogeneity but limits the generalizability of the findings to male patients with FM. Third, the study was conducted at a single center with a relatively modest sample size, and the CART classification model was not externally validated. Fourth, all psychological and sleep-related variables were assessed using self-report questionnaires, making response bias and shared-method variance unavoidable. In addition, medication use (including antidepressants, anxiolytics, analgesics, and hypnotics), menopausal status, psychiatric history, and other potentially important clinical confounders were not systematically evaluated and therefore could not be incorporated into the analytical models. These unmeasured factors may have influenced both psychological symptoms and fibromyalgia severity. Finally, although the proposed path model was theoretically informed, additional longitudinal, multicenter, and mechanistic studies are needed to confirm the observed associations. Accordingly, the present findings provide a framework for future investigation but should not be interpreted as definitive evidence regarding disease mechanisms.

## 5. Conclusions

In conclusion, depressive symptoms, sleep disturbance, and anxiety symptoms were consistently the variables most closely associated with fibromyalgia severity across multiple analytical methods. In contrast, the selected biological variables did not demonstrate consistent independent associations with disease severity, although TSH and 25-hydroxyvitamin D showed distinct indirect relationships with psychological variables.

By integrating multiple linear regression, Classification and Regression Tree (CART) analysis, and path analysis, this study provides a comprehensive perspective on the relationships among psychological symptoms, selected biological variables, and fibromyalgia severity. These complementary analytical methods do not support causal inferences but improve the interpretation of complex clinical associations within a biopsychosocial framework.

Overall, the findings highlight the importance of comprehensive assessment of psychological symptoms and sleep disturbance in women with FM and provide a framework for future longitudinal and multicenter studies to validate the observed classification patterns and clarify the temporal relationships underlying fibromyalgia.

## Figures and Tables

**Table 1:** Comparison of depressive symptoms, anxiety symptoms, and sleep disturbance between women with fibromyalgia and healthy controls.

Variable	FM (*n* = 102) Mean ± SD	Control (*n* = 53) Mean ± SD	*p*-Value	Cohen’s d
BDI	16.59 ± 9.70	3.42 ± 4.58	<0.001	1.58
BAI	16.34 ± 12.32	4.21 ± 4.06	<0.001	1.18
JSS	13.42 ± 5.42	3.08 ± 3.90	<0.001	2.09

Abbreviations: FM, fibromyalgia; BDI, Beck Depression Inventory; BAI, Beck Anxiety Inventory; JSS, Jenkins Sleep Scale. Cohen’s d values of approximately 0.20, 0.50, and 0.80 were interpreted as small, medium, and large effect sizes, respectively.

**Table 2:** Comparison of hematologic and biochemical parameters between the fibromyalgia and control groups.

Variable	Unit	FM (*n* = 102) Mean ± SD	Control (*n* = 53) Mean ± SD	*p*-Value
WBC	×10^9^/L	6.84 ± 1.72	7.09 ± 1.94	0.405
RBC	×10^12^/L	4.35 ± 0.48	4.77 ± 0.39	<0.001
HGB	g/dL	12.56 ± 1.37	12.93 ± 1.39	0.116
PLT	×10^9^/L	280.74 ± 70.59	333.64 ± 53.00	0.146
TSH	µIU/mL	2.49 ± 3.53	1.83 ± 1.24	0.191
fT4	ng/dL	1.03 ± 0.21	1.15 ± 0.20	0.002
Ferritin	ng/mL	19.43 ± 19.48	22.94 ± 21.21	0.304
Vitamin D	nmol/L	47.60 ± 25.83	48.77 ± 28.91	0.797
ESR	mm/h	11.39 ± 6.33	13.55 ± 9.20	0.089
Vitamin B12	pg/mL	389.56 ± 597.09	330.83 ± 79.46	0.478

Abbreviations: FM, fibromyalgia; WBC, white blood cell count; RBC, red blood cell count; HGB, hemoglobin; PLT, platelet count; TSH, thyroid-stimulating hormone; fT4, free thyroxine; ESR, erythrocyte sedimentation rate.

**Table 3:** Classification performance of the final CART model.

Performance Metric	Value (95% CI)
Overall accuracy	93.5% (88.5–96.5)
Sensitivity	90.2% (82.9–94.6)
Specificity	100.0% (93.2–100.0)
Positive predictive value (PPV)	100.0% (96.0–100.0)
Negative predictive value (NPV)	84.1% (73.2–91.1)
Area under the ROC curve (AUC)	0.951

Abbreviations: CART, Classification and Regression Tree; PPV, positive predictive value; NPV, negative predictive value; CI, confidence interval.

**Table 4:** Confusion matrix of the final CART model.

	Predicted FM	Predicted Healthy Control	Total
Actual FM	92	10	102
Actual Healthy Control	0	53	53
Total	92	63	155

Overall accuracy: 93.5%; sensitivity: 90.2%; specificity: 100.0%. Abbreviations: FM, fibromyalgia.

**Table 5:** Goodness-of-fit indices for the path analysis model.

Fit Index	Observed Value	Recommended Threshold
χ^2^	4.29	—
Degrees of freedom (df)	4	—
χ^2^/df	1.07	<3.0
*p* value	0.368	>0.05
Comparative Fit Index (CFI)	0.997	≥0.90
Goodness-of-Fit Index (GFI)	0.999	≥0.90
Standardized Root Mean Square Residual (SRMR)	0.028	≤0.08
Root Mean Square Error of Approximation (RMSEA)	0.027	≤0.08
RMSEA 95% CI	0.00–0.15	—

Abbreviations: CFI, Comparative Fit Index; GFI, Goodness-of-Fit Index; SRMR, Standardized Root Mean Square Residual; RMSEA, Root Mean Square Error of Approximation; CI, confidence interval. Model fit was evaluated using multiple complementary goodness-of-fit indices. Lower values of χ^2^/df, SRMR, and RMSEA and higher values of CFI and GFI indicate better model fit.

**Table 6:** Standardized path coefficients of the final model.

Path	B	SE	Standardized β	95% CI	*p* Value
TSH → BDI	0.486	0.260	0.177	−0.030 to 1.002	0.065
JSS → BDI	0.513	0.170	0.287	0.177 to 0.850	0.003
Vitamin D → BAI	−0.117	0.046	−0.246	−0.209 to −0.025	0.013
BDI → FIQ	0.574	0.119	0.399	0.338 to 0.810	<0.001
JSS → FIQ	0.930	0.192	0.362	0.550 to 1.311	<0.001
BAI → FIQ	0.254	0.089	0.224	0.077 to 0.431	0.005

Abbreviations: TSH, thyroid-stimulating hormone; BDI, Beck Depression Inventory; BAI, Beck Anxiety Inventory; JSS, Jenkins Sleep Scale; FIQ, Fibromyalgia Impact Questionnaire; SE, standard error; CI, confidence interval. Unstandardized regression coefficients (B), standardized path coefficients (β), standard errors (SEs), 95% confidence intervals (CIs), and corresponding *p* values are shown for each direct path.

**Table 7:** Bootstrap estimates of indirect effects in the final path analysis model.

Indirect Path	Bootstrap Estimate	Bootstrap SE	95% Bootstrap CI	Statistical Significance
TSH → BDI → FIQ	0.238	0.191	−0.264 to 0.525	Not significant
JSS → BDI → FIQ	0.294	0.104	0.108 to 0.516	Significant
Vitamin D → BAI → FIQ	−0.029	0.013	−0.057 to −0.007	Significant

Abbreviations: TSH, thyroid-stimulating hormone; BDI, Beck Depression Inventory; BAI, Beck Anxiety Inventory; JSS, Jenkins Sleep Scale; FIQ, Fibromyalgia Impact Questionnaire; SE, standard error; CI, confidence interval.

## Data Availability

The datasets generated and/or analyzed during the current study are available from the corresponding author upon reasonable request.

## Abbreviations

The following abbreviations are used in this manuscript:

ACR	American College of Rheumatology
AUC	Area Under the Receiver Operating Characteristic Curve
BAI	Beck Anxiety Inventory
BDI	Beck Depression Inventory
BMI	Body Mass Index
CART	Classification and Regression Tree
CFI	Comparative Fit Index
CI	Confidence Interval
FM	Fibromyalgia
FIQ	Fibromyalgia Impact Questionnaire
fT4	Free Thyroxine
GFI	Goodness-of-Fit Index
HC	Healthy Control
JSS	Jenkins Sleep Scale
NPV	Negative Predictive Value
PPV	Positive Predictive Value
RBC	Red Blood Cell
RMSEA	Root Mean Square Error of Approximation
SEM	Structural Equation Modeling
SRMR	Standardized Root Mean Square Residual
TSH	Thyroid-Stimulating Hormone
25(OH)D	25-Hydroxyvitamin D
